# Ultrasonographic Assessment of Upper Airway Structures in Adult Obstructive Sleep Apnea: A Systematic Review

**DOI:** 10.3390/jcm15093213

**Published:** 2026-04-23

**Authors:** Cristina Rodríguez Alcalá, Carlos O’Connor Reina, Eduardo Javier Correa, Laura Rodríguez Alcalá, José María Ignacio García, Francisco Javier Gómez Jiménez

**Affiliations:** 1 Department of Medicine, University of Granada, 18071 Granada, Spain; 2 Department of Otolaryngology, Quirónsalud Marbella Hospital, 29602 Marbella, Spain; 3 Department of Pulmonology, Quirónsalud Marbella Hospital, 29602 Marbella, Spain

**Keywords:** obstructive sleep apnea, ultrasonography, upper airway, tongue thickness, elastography, airway phenotyping, drug-induced sleep endoscopy, myofunctional therapy

## Abstract

**Background**: Ultrasonography (US) has emerged as a non-invasive method for anatomical and functional evaluation of upper airway structures in adult obstructive sleep apnea (OSA). However, its role in severity stratification, dynamic assessment, elastographic characterization, and therapeutic monitoring remain to be investigated. **Background/Objectives**: The goal herein is thus to systematically review and synthesize available evidence on US assessment in adults with OSA, including structural parameters, dynamic measurements, correlation with the apnea–hypopnea index (AHI), integration with artificial intelligence, and evaluation of myofunctional therapy outcomes. **Methods**: A PRISMA-compliant systematic review of 19 studies (2007–2025) was conducted, evaluating US in adult patients with polysomnography-diagnosed OSA. Observational, pilot, case–control, and exploratory studies were included. Risk of bias was assessed using the National Institutes of Health Quality Assessment Tool for observational studies. Due to methodological heterogeneity, a structured qualitative meta-analytic synthesis was performed. **Results**: The tongue base was the most frequently studied structure. Increased tongue thickness, area, and stiffness were consistently associated with higher AHI. Elastography revealed increased intrinsic rigidity in patients with OSA. Dynamic US correlated with drug-induced sleep endoscopy findings and hyoid displacement. Machine learning integration improved severity prediction. A single study evaluated anatomical changes following myofunctional therapy, representing a nascent research area. US may become a complementary, non-invasive tool for anatomical and functional assessment of upper airway structures in adult OSA. **Conclusions**: Further standardization of acquisition protocols and well-designed longitudinal studies are needed to clarify the clinical role of US in phenotyping and therapeutic monitoring.

## 1. Introduction

Obstructive sleep apnea (OSA) is characterized by repetitive upper airway collapse during sleep, resulting in intermittent hypoxia and sleep fragmentation [1]. Although polysomnography (PSG) is the diagnostic gold standard for sleep disorders [2], it provides limited insight into the underlying pathophysiological mechanisms, as it enables neither anatomical nor functional direct assessment of the upper airway during obstructive events, either at baseline or throughout the dynamic process of airway collapse [3]. Despite significant advances in diagnosis and management, understanding these underlying mechanisms remains a major clinical and research challenge.

Upper airway collapse in OSA is a complex, multilevel phenomenon involving interactions among the tongue base, soft palate, lateral pharyngeal walls, and hyoid-associated musculature, which are influenced by neuromuscular control, tissue compliance, and craniofacial morphology [4]. Conventional imaging methods like magnetic resonance imaging and computed tomography (CT) provide detailed anatomical visualizations; nevertheless, their clinical applications are constrained by cost, limited accessibility, radiation exposure (in the case of CT), and restricted capability for dynamic or bedside assessments under physiological conditions [5].

Ultrasonography (US) has emerged as a promising alternative imaging approach for upper airway evaluation. The theoretical and practical advantages of US include its non-invasive nature, portability, absence of ionizing radiation, relatively low cost, bedside applicability, and dynamic real-time imaging [6]. Additionally, recent technological advances have expanded its potential applications beyond static morphology, incorporating elastographic techniques for tissue stiffness evaluation and facilitating integration with artificial intelligence-based analytic models. These features position US as a potentially valuable tool for OSA phenotyping, guiding therapeutic decision-making, and monitoring treatment responses [7].

The objective of this systematic review is to summarize and critically appraise the current evidence on the use of US for the assessment of upper airway structures and for monitoring therapeutic interventions in adults with OSA.

## 2. Materials and Methods

We developed a systematic review protocol in accordance with PRISMA 2020 [8] and registered it prospectively in the PROSPERO database (CRD420261290459) on 29 January 2026. No amendments were made to the protocol after registration. A comprehensive literature search was conducted between January and March 2026 across the following electronic databases: PubMed (MEDLINE), Scopus, EMBASE, Web of Science, and the Cochrane Library. The final search was performed on 2 March 2026, ensuring inclusion of the most recent studies published up to that date. Search strategies were developed using Medical Subject Headings (MeSH) and database-specific controlled vocabulary, combined with relevant free-text terms, as detailed in Table 1. Filters were applied to restrict results to studies published in English and conducted in human adults (≥18 years) with OSA. In addition, clinical trial registries were searched through ClinicalTrials.gov to identify ongoing or unpublished studies. Reference lists of all included articles and relevant reviews were also manually screened for additional eligible studies (i.e., backward citation searching).

The full search strategy and corresponding results are provided in Supplementary Materials.

Following the initial search, all retrieved records were deduplicated using the Evidence Review Accelerator (TERA^®^) [9]. Study screening and selection were conducted in two phases. First, titles and abstracts of all retrieved records were independently screened by two reviewers using the Rayyan web application (latest version, Qatar Computing Research Institute, Doha, Qatar) [10] to identify potentially eligible studies. In the second phase, the full texts of the selected articles were independently assessed for inclusion by the same two reviewers. Any disagreements at either stage were resolved through discussion and consensus, or, when necessary, adjudicated by a third independent reviewer. The methodological quality of the included studies was assessed using a domain-based risk of bias approach adapted for observational studies.

### Eligibility Criteria

Original research studies evaluating the use of US for the assessment of upper airway structures and/or monitoring of therapeutic interventions in adults with OSA were included. Eligible study designs comprised cross-sectional, case–control, cohort, and interventional studies. Case series with a minimum sample size of ≥10 participants were also considered. Reviews, editorials, conference abstracts, letters, and case reports were excluded. Only studies published in English were included, with no restrictions regarding the year of publication.

Studies enrolling adults (≥18 years) diagnosed with OSA according to standard sleep study criteria (PSG or respiratory polygraphy, based on American Academy of Sleep Medicine definitions) [11] were eligible, whereas studies exclusively involving pediatric populations were excluded.

US had to be used as the primary imaging modality for baseline anatomical assessment and/or for monitoring treatment-related anatomical changes in upper airway structures. Studies focusing exclusively on non-upper airway US applications were not considered.

Studies with or without comparator groups were eligible, as no specific comparator was required for inclusion. Finally, included studies were required to report US-derived findings related to upper airway structures, such as anatomical measurements, dynamic functional changes, or treatment-related structural adaptations relevant to OSA.

Due to substantial methodological and clinical heterogeneity among the included studies, a quantitative meta-analysis was not performed. Heterogeneity was observed in US acquisition protocols (submental vs. lateral approaches), patient positioning (awake, natural sleep, or drug-induced sleep endoscopy [DISE] conditions), technical parameters, and operator experience. Additionally, outcome definitions varied considerably, including tongue thickness, cross-sectional area, estimated volume, elastographic stiffness, and dynamic motion indices. Clinical heterogeneity was also present, with differences in body mass index (BMI) distribution, OSA severity classification, and study design. Given these variations, statistical pooling was deemed inappropriate, and a structured qualitative synthesis was instead conducted to preserve methodological rigour and interpretative validity.

## 3. Results

**Figure 1:**
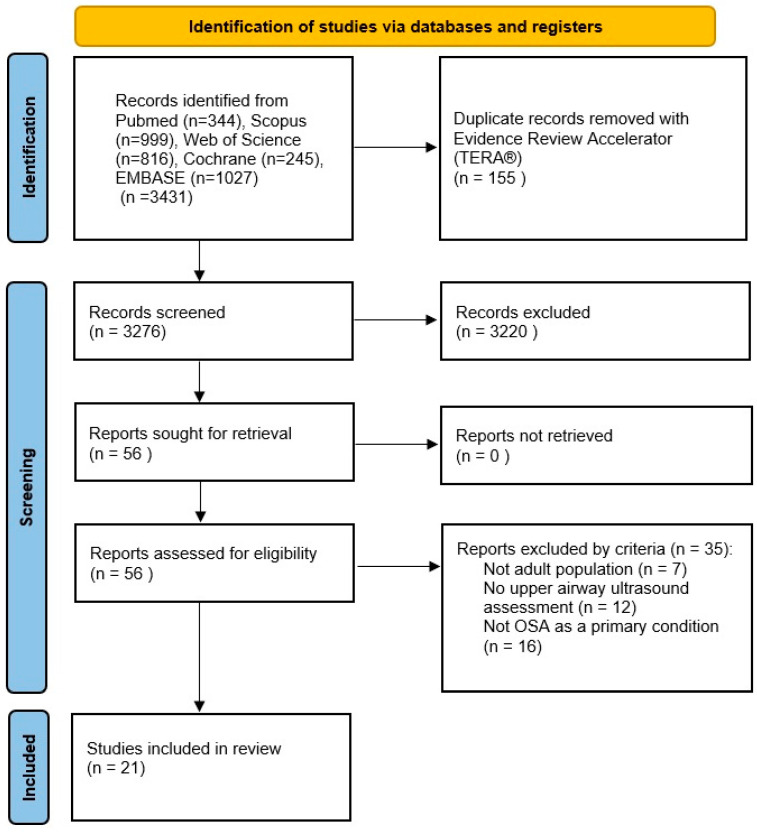
PRISMA flow diagram of the study selection process.

After screening and eligibility assessment, 21 studies were included in the qualitative synthesis (Figure 1).

Flowchart showing the identification, screening, eligibility assessment, and inclusion of studies evaluating ultrasonographic assessment of upper airway structures in adult patients with obstructive sleep apnea.

To improve clarity, the included studies were categorized according to their primary methodological focus, as shown in Table 2.

The included studies were published between 2007 and 2025 and primarily involve adult patients diagnosed with OSA by PSG. Most were observational and cross-sectional in design. Only one prospective controlled clinical study evaluated longitudinal US changes following therapeutic intervention.

### 3.1. Morphological Assessment

The earliest US-based assessments of lingual morphology were reported by Liu et al. [12] and Lahav et al. [13], both demonstrating that increased tongue thickness correlated with the apnea–hypopnea index (AHI). Subsequent studies by Chen et al. [14] and Chien et al. [15] confirm that tongue thickness is greater in patients with more severe OSA.

Dynamic thickness measurements during natural sleep or DISE was explored by Weng et al. [16,21] who report altered tongue motion patterns in patients with OSA. Manlises et al. [17] further show that the tongue cross-sectional area is increased in severe OSA.

Global upper airway dimensions were evaluated by Hussein et al. [18] and Chen et al. [19], both demonstrating correlations between US-derived measurements and AHI. Similarly, Saha et al. [19] report that reduced pharyngeal dimensions were associated with higher BMI and OSA severity.

More recent studies reinforce these findings. Bosschieter et al. [6] report increased tongue volume in severe OSA using standardized imaging protocols. Terawatpothong et al. [31] validate a submental US protocol for OSA severity prediction. Ravindranath et al. [20] propose a point-of-care US-based airway index that correlates with screening questionnaires and AHI values.

### 3.2. Dynamic Assessment and DISE Correlation

The relations between US findings and endoscopic collapse patterns were explored by Abuan et al. [22], who demonstrate that US could support tongue base evaluation during DISE. Hyoid displacement and its physiological determinants were examined by Parekh et al. [22] and later expanded by Parekh et al. [23], showing that caudal hyoid motion correlates with tongue collapse during DISE.

Dynamic US reliability was specifically addressed by Saha et al. [30] and Chu et al. [26], the latter focusing on elastography reproducibility.

### 3.3. Elastography

Lingual stiffness assessment using shear wave elastography was investigated by Chang et al. [25] and Chu et al. [26]. Both report increased intrinsic tongue stiffness in patients with OSA compared with controls, with acceptable intra- and inter-observer agreements.

### 3.4. Artificial Intelligence Applications

Machine learning-assisted severity prediction models were introduced by Ontimare et al. [27], who demonstrate improved discrimination of OSA severity using US-derived parameters. This approach was further expanded by Jones et al. [28], who combine US data and machine learning to predict DISE-observed collapse patterns.

### 3.5. Longitudinal and Interventional Evidence

Only one completed prospective controlled clinical study evaluates US-detected anatomical changes following therapy. Rodríguez-Alcalá et al. [29] investigated the impacts of myofunctional therapy on tongue morphology and upper airway parameters, reporting reductions in tongue volume, along with improvements in tongue strength and airway dynamics. An earlier protocol published by the same group [32] describes the study design and preliminary data but was not analyzed separately due to incomplete outcomes at that stage.

To provide a comprehensive overview of the methodological characteristics of the included studies, Table 3 summarizes key aspects of the study design, diagnostic criteria, ultrasound evaluation, and examination conditions.

### 3.6. Risk of Bias Assessment

The risk of bias of the included studies was assessed across several domains, including participant selection, predictor measurement, outcome assessment, confounding, incomplete data, statistical analysis, and selective reporting. Detailed results of the risk of bias assessment are presented in Table 4.

The risk of bias assessment indicates an overall low risk of bias across most methodological domains. Most studies used objective ultrasonographic measurements to evaluate anatomical or functional characteristics of the upper airway, including tongue thickness, tongue stiffness, airway dimensions, and hyoid motion. These imaging-based measurements reduce subjectivity and contribute to methodological robustness.

Similarly, the assessment of obstructive sleep apnea severity is considered to have a low risk of bias in the majority of studies, as most investigations relied on standard diagnostic methods such as polysomnography or validated clinical indices of apnea severity.

However, moderate concerns were identified in the domains of participant selection and confounding factors. All studies were observational in design and typically recruited patients from clinical populations referred to for sleep evaluation. This may introduce selection bias and limit the generalizability of findings to broader populations. Additionally, several potential confounders—such as body mass index, age, sex, and craniofacial morphology—may influence upper airway anatomy and ultrasound measurements. These factors were not consistently controlled across studies.

**Figure 2:**
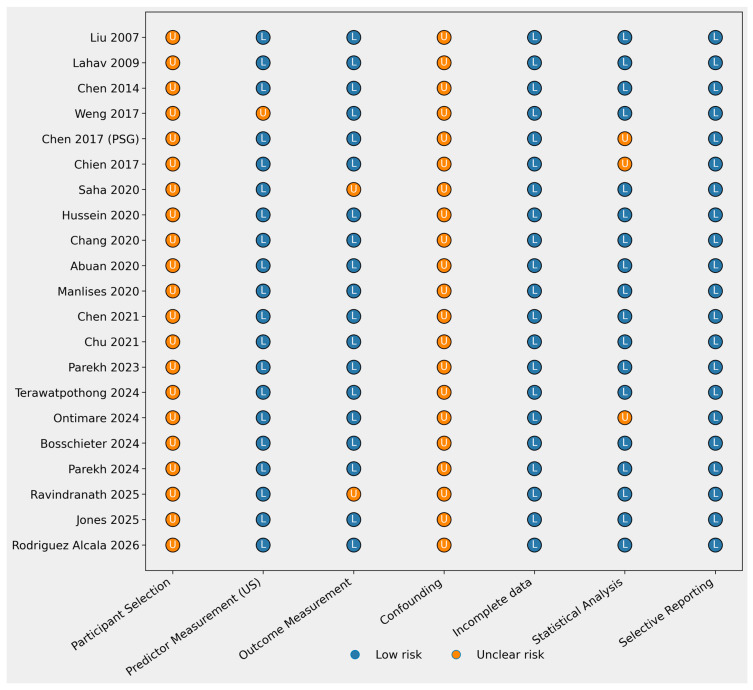
Traffic light plot of risk of bias [6,12,13,14,15,16,17,18,19,20,21,22,23,24,25,26,27,28,29,30,31].

The risk of bias related to incomplete outcome data and selective reporting is low in nearly all studies, as most reports provided complete datasets and clearly described their statistical analyses (Figure 2).

## 4. Discussion

This systematic review of 21 studies explores the use of US in adult OSA. What became evident from this review is that US has been consistently used to characterize tongue-related parameters, and that these measurements are frequently associated with OSA severity. Simultaneously, this body of evidence is currently fragmented, methodologically diverse, and largely observational.

Most of the included studies focus on the tongue base. Across different populations and protocols, increased tongue thickness, cross-sectional area, and estimated volume tend to correlate with higher AHI values [13]. Although the absolute measurements varied among studies, the direction of the association was remarkably consistent. This supports the idea that tongue morphology plays a central role in airway collapsibility [32,33].

However, US offers something beyond static anatomy. Several studies evaluated dynamic tongue motion either during natural sleep or under DISE conditions [16,20]. These investigations suggest that OSA is not simply a problem of enlarged structures, but of altered movement patterns and insufficient stabilization of the airway during respiratory cycles. In this sense, US begins to bridge the gap between structural imaging and functional assessment [28].

Elastography adds another layer of complexity. Increased tongue stiffness has been described in patients with OSA, which may initially seem paradoxical in a disorder traditionally associated with hypotonia. Yet stiffness should not be equated with effective neuromuscular control [24]. Chronic remodelling, fatty infiltration, or compensatory overactivation may explain these findings. What matters clinically is not stiffness in isolation, but whether the muscle can maintain airway patency during sleep [3].

An additional emerging field where ultrasonography may have clinical relevance is hypoglossal nerve stimulation (HGNS). Recent studies have explored the use of ultrasound to assess tongue motion and muscle response during upper airway stimulation. For instance, Fleury et al. [34] demonstrate the feasibility of ultrasound in evaluating functional changes associated with HGNS therapy, while Hofauer et al. [35] report sonographic visualization of tongue motion patterns during stimulation. These findings suggest that US could provide a non-invasive and dynamic tool to complement current evaluation methods in patients undergoing HGNS, although evidence remains limited and requires further validation.

The integration of machine learning into US analysis represents a more recent development. Preliminary models show that combining multiple US-derived parameters improves the prediction of OSA severity and even DISE-observed collapse patterns. While promising, these approaches remain exploratory and require external validation before they can be translated into routine clinical use [36].

One of the most relevant observations that emerged from this review is the scarcity of longitudinal data. Nearly all published studies are cross-sectional and focus on the associations between US-derived measurements and OSA severity. To date, only one prospective controlled study systematically evaluated treatment-related anatomical changes using US [29,32].

**Figure 3:**
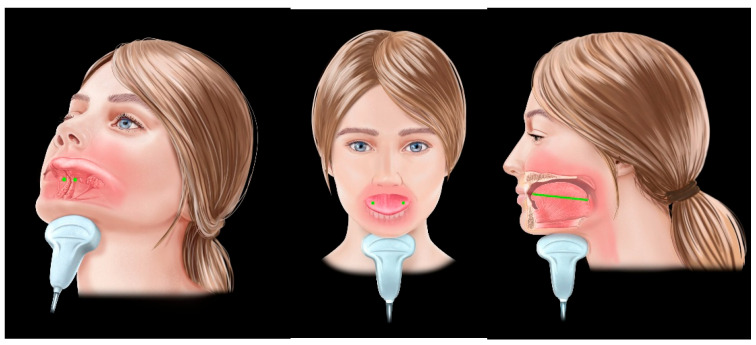
Measurements of tongue thickness, volume, and inter-lingual distance.

In that study, Rodríguez-Alcalá et al. demonstrate that telemedicine-delivered myofunctional therapy is associated with measurable reductions in tongue volume and inter-lingual arterial distance, accompanied by improvements in tongue strength and collapse patterns during DISE. Notably, these structural modifications occur without significant changes in BMI, suggesting that the observed remodelling is functionally mediated rather than weight-related (Figure 3).

A relevant aspect emerging from the literature is the comparison between ultrasonography and established imaging modalities such as drug-induced sleep endoscopy (DISE) and magnetic resonance imaging (MRI) [1]. While DISE remains the gold standard for dynamic evaluation of airway collapse, ultrasonography offers a non-invasive and bedside alternative capable of capturing real-time structural and functional changes. Several studies included in this review demonstrate correlations between ultrasonographic findings and DISE-observed collapse patterns, particularly at the level of the tongue base and hyoid displacement [17,21,28,29,32]. Compared to MRI, US lacks comprehensive anatomical coverage but provides superior accessibility and feasibility for repeated assessments. Therefore, US should be considered complementary rather than a replacement for these techniques.

Methodologically, this study is notable for integrating serial US measurements, objective muscle strength assessment, endoscopic findings, and polysomnographic outcomes within the same cohort. It therefore represents the first controlled attempt to move US beyond static correlation, towards longitudinal monitoring of airway adaptation.

Nevertheless, these findings should be interpreted within the context of a single-centre design and require confirmation in independent and larger cohorts.

The current, cumulative evidence positions US as a complementary modality. It cannot replace PSG, nor does it substitute for endoscopic evaluation. However, it offers a non-invasive, bedside method to explore structural and dynamic characteristics of the upper airway. Its accessibility and safety profile make it particularly attractive for outpatient settings and repeated measurements over time [37].

The main challenge moving forward will be standardization. Differences in probe positioning, patient posture, measurement definitions, and operator expertise limit comparability across studies. Without consensus protocols, it will be difficult to establish normative values or integrate US into phenotyping algorithms [23].

Despite these limitations, the overall consistency of associations between tongue-related US parameters and OSA severity across independent cohorts suggests that these are not spurious findings. Rather, it reflects a reproducible anatomical and functional pattern that merits further investigation [38].

Future research should focus on multicentre studies with harmonized acquisition protocols and clearly defined outcome measures. Only then will it be possible to determine whether US can evolve from a research tool into a clinically meaningful component of OSA assessment and follow-up.

### Limitations

Despite its strengths, several limitations of this systematic review must be acknowledged.

First, the overall level of evidence remains predominantly observational. Most included studies are cross-sectional in design, limiting causal inference and preventing definitive conclusions regarding the predictive, mechanistic, or prognostic value of US-derived parameters in OSA.

Second, substantial methodological heterogeneity was identified across studies. Differences were observed in US acquisition protocols (submental vs. lateral approaches), patient positioning (awake, natural sleep, or DISE conditions), technical settings, operator expertise, and the specific anatomical or functional parameters assessed (thickness, cross-sectional area, estimated volume, stiffness, dynamic indices). This heterogeneity precludes quantitative meta-analysis and limits direct inter-study comparability.

Third, many studies were conducted in single-centre settings with relatively small sample sizes, which may have introduced selection bias and reduced generalizability. Moreover, US is inherently operator-dependent. Although several studies report acceptable intra- and inter-rater reliability, standardized acquisition protocols, calibration procedures, and reproducibility frameworks are not yet universally established.

Fourth, longitudinal and interventional evidence remains extremely limited. Only one prospective controlled study systematically evaluated US-detected anatomical changes following therapeutic intervention. Therefore, conclusions regarding the role of US as a monitoring biomarker for treatment response must be interpreted cautiously and considered hypothesis-generating rather than confirmatory.

Fifth, studies incorporating artificial intelligence-based predictive models were based on relatively small datasets and lack independent external validation cohorts. Consequently, their clinical applicability and reproducibility remain investigational.

Finally, the possibility of publication bias cannot be excluded. Studies demonstrating positive associations between US parameters and OSA severity may be over-represented in the literature, whereas negative or inconclusive findings may be under-reported.

Despite these limitations, the consistency of associations between US-derived tongue characteristics and OSA severity across independent cohorts, together with emerging interventional data, support continued investigation and the development of standardized protocols in this field.

## 5. Conclusions

US of the tongue and upper airway may represent a practical, non-invasive approach to assessing anatomical and functional features in adults with OSA. Across the available studies, tongue-related measurements such as thickness, cross-sectional area, estimated volume, and stiffness are frequently associated with OSA presence and severity.

Most of the current evidence, however, remains observational and methodologically heterogeneous. Longitudinal data are limited, and only one prospective controlled study has evaluated treatment-related anatomical changes using US. As such, the use of US as a tool for monitoring therapeutic response should be interpreted with caution.

Further prospective studies using standardized acquisition protocols and reproducible measurement strategies are needed to better define the clinical role of US in phenotyping, treatment selection, and follow-up in OSA.

## Figures and Tables

**Table 1:** MeSH terms.

MeSH Terms	
AND	
ultrasound	sleep apnea
ultrasounds	sleep apnea hypopnea syndrome
ultrasound diagnostic	sleep apnea syndrome, obstructive
ultrasound examination	sleep apnea, obstructive
ultrasound imaging	sleep apneas
ultrasound imaging, Doppler	sleep apneas, obstructive
ultrasound, diagnostic	sleep disordered breathing
ultrasounds, diagnostic	snoring
ultrasound, Doppler	

**Table 2:** Classification of included studies by primary focus (*n* = 21).

Category	Number of Studies	Included Studies
Morphological assessment	10	Liu et al., 2007 [12]; Lahav et al., 2009 [13]; Chen et al., 2014 [14]; Chien et al., 2017 [15]; Chen et al., 2017 (PSG) [16]; Manlises et al., 2020 [17]; Hussein et al., 2020 [18]; Chen et al., 2021 [19]; Bosschieter et al., 2024 [6]; Ravindranath et al., 2025 [20].
Dynamic assessment	4	Weng et al., 2017 [21]; Abuan et al., 2020 [22]; Parekh et al., 2023 [23]; Parekh et al., 2024 [24].
Elastography	2	Chang et al., 2020 [25]; Chu et al., 2021 [26].
Artificial intelligence	2	Ontimare et al., 2024 [27]; Jones et al., 2025 [28].
Longitudinal/interventional	1	Rodríguez-Alcalá et al., 2026 [29].
Mixed approaches	2	Saha et al., 2020 [30]; Terawatpothong et al., 2024 [31].

Note: Each study was classified according to its primary methodological focus to avoid duplication across categories.

**Table 3:** Characteristics of the studies included in the systematic review evaluating ultrasonographic assessment of upper airway structures in adults with obstructive sleep apnea.

Authors	Study Type	OSA Diagnosis	Ultrasound Evaluation	Condition
Lahav et al., 2009 [13]	Observational	PSG	Tongue base thickness	Awake/Supine
Liu et al., 2007 [12]	Case–Control	PSG	Parapharyngeal wall thickness	Awake/Supine
Chen et al., 2014 [14]	Observational	PSG	Tongue base dynamic thickness	Awake/Supine
Chien et al., 2017 [15]	Observational	PSG	Tongue dynamic movement	Awake/Supine
Weng et al., 2017 [21]	Pilot	PSG	Tongue base dynamic thickness	Natural sleep or DISE/Supine
Chen et al., 2017 [16]	Pilot	PSG	Tongue base dynamic thickness	Natural sleep/Supine
Manlises et al., 2020 [17]	Observational	PSG	Tongue area	Awake/Supine
Chang et al., 2020 [25]	Observational	PSG	Tongue elastography	Awake/Supine
Abuan et al., 2020 [22]	Observational	PSG + DISE	Tongue thickness + dynamic movement	Awake/Supine
Chu et al., 2021 [26]	Methodologic	PSG	Tongue elastography	Awake/Supine
Hussein et al., 2020 [18]	Case–control	PSG	Upper airway dimensions	Awake/Supine
Chen et al., 2021 [19]	Observational	PSG	Upper airway dimensions	Awake/Supine
Bosschieter et al., 2024 [6]	Observational	PSG	Tongue area and dynamic movement	Awake/Supine
Ontimare et al., 2024 [27]	Observational + Machine Learning	PSG	Tongue dynamic movement	Awake/Supine
Parekh et al., 2023 [23]	Observational	PSG + DISE	Hyoid displacement	DISE/Supine
Parekh et al., 2024 [24]	Observational	PSG +DISE	Hyoid displacement	DISE/Supine
Saha et al., 2020 [30]	Observational	PSG	Pharyngeal area	Awake/Supine
Terawatpothong et al., 2024 [31]	Observational	Questionnaires/PSG	Upper airway dimensions	Awake/Supine
Ravindranath et al., 2025 [20]	Observational	Questionnaires/PSG	Upper airway dimensions	Awake/Supine
Jones et al., 2025 [28]	Observational	PSG + DISE	Upper airway dimensions	DISE/Supine
Rodriguez Alcala et al., 2026 [29]	Prospective controlled	PSG + DISE	Tongue/Upper airway baseline parameters and morphological changes	Awake + DISE/Supine

PSG: polysomnography; DISE: drug-induced sleep endoscopy; US: ultrasonography. Note: Studies are presented in chronological order. Ultrasound evaluation refers to the primary anatomical or functional parameter assessed in each study.

**Table 4:** Risk of bias assessment of the included studies across methodological domains.

Study	Participant Selection	Predictor Measurement (US)	Outcome Measurement	Confounding	Incomplete Data	Statistical Analysis	Selective Reporting
Liu 2007 [12]	Unclear	Low	Low	Unclear	Low	Low	Low
Lahav 2009 [13]	Unclear	Low	Low	Unclear	Low	Low	Low
Chen 2014 [14]	Unclear	Low	Low	Unclear	Low	Low	Low
Weng 2017 [21]	Unclear	Unclear	Low	Unclear	Low	Low	Low
Chen 2017 (PSG) [16]	Unclear	Low	Low	Unclear	Low	Unclear	Low
Chien 2017 [15]	Unclear	Low	Low	Unclear	Low	Unclear	Low
Saha 2020 [30]	Unclear	Low	Unclear	Unclear	Low	Low	Low
Hussein 2020 [18]	Unclear	Low	Low	Unclear	Low	Low	Low
Chang 2020 [25]	Unclear	Low	Low	Unclear	Low	Low	Low
Abuan 2020 [22]	Unclear	Low	Low	Unclear	Low	Low	Low
Manlises 2020 [17]	Unclear	Low	Low	Unclear	Low	Low	Low
Chen 2021 [19]	Unclear	Low	Low	Unclear	Low	Low	Low
Chu 2021 [26]	Unclear	Low	Low	Unclear	Low	Low	Low
Parekh 2023 [23]	Unclear	Low	Low	Unclear	Low	Low	Low
Terawatpothong 2024 [31]	Unclear	Low	Low	Unclear	Low	Low	Low
Ontimare 2024 [27]	Unclear	Low	Low	Unclear	Low	Unclear	Low
Bosschieter 2024 [6]	Unclear	Low	Low	Unclear	Low	Low	Low
Parekh 2024 [24]	Unclear	Low	Low	Unclear	Low	Low	Low
Jones 2025 [28]	Unclear	Low	Low	Unclear	Low	Low	Low
Ravindranath 2025 [20]	Unclear	Low	Unclear	Unclear	Low	Low	Low
Rodriguez Alcala 2026 [29]	Unclear	Low	Low	Unclear	Low	Low	Low

## Data Availability

The data supporting the findings of this study are available within the article and its Supplementary Materials.

## Supplementary Materials

The following supporting information can be downloaded at: https://www.mdpi.com/article/10.3390/jcm15093213/s1, PRISMA 2020 Checklist.

## Abbreviations

The following abbreviations are used in this manuscript:

AHI	Apnea–Hypopnea Index
AI	Artificial Intelligence
AOS	Apnea Obstructiva del Sueño/Obstructive Sleep Apnea
BMI	Body Mass Index
CNN	Convolutional Neural Network
CPAP	Continuous Positive Airway Pressure
DISE	Drug-Induced Sleep Endoscopy
EEG	Electroencephalography
EMG	Electromyography
EOG	Electrooculography
GRU	Gated Recurrent Unit
ML	Machine Learning
MRI	Magnetic Resonance Imaging
NREM	Non-Rapid Eye Movement Sleep
OMT	Orofacial Myofunctional Therapy
OSA	Obstructive Sleep Apnea
PSG	Polysomnography
PRISMA	Preferred Reporting Items for Systematic Reviews and Meta-Analyses
REM	Rapid Eye Movement Sleep
RNN	Recurrent Neural Network
SDB	Sleep-Disordered Breathing
SpO_2_	Peripheral Oxygen Saturation
US	Ultrasound

## References

[1] Schwab R.J., Pasirstein M., Pierson R., Mackley A., Hachadoorian R., Arens R., Maislin G., Pack A.I. (2003). Identification of upper airway anatomic risk factors for obstructive sleep apnea with volumetric magnetic resonance imaging. Am. J. Respir. Crit. Care Med..

[2] Weaver E.M., Woodson B.T., Steward D.L. (2005). Polysomnography indexes are discordant with quality of life, symptoms, and reaction times in sleep apnea patients. Otolaryngol. Head Neck Surg..

[3] O’Connor-Reina C., Baptista P., Plaza G. (2026). Reconsidering the hypotonic phenotype: From static anatomy to dynamic airway function in obstructive sleep apnea. Sleep Med. Rev..

[4] Mesti J.J., Cahali M.B. (2012). Evolution of swallowing in lateral pharyngoplasty with stylopharyngeal muscle preservation. Braz. J. Otorhinolaryngol..

[5] Harvey R., O’BRien L., Aronovich S., Shelgikar A., Hoff P., Palmisano J., Stanley J. (2017). Friedman tongue position and cone beam computed tomography in patients with obstructive sleep apnea. Laryngoscope Investig. Otolaryngol..

[6] Bosschieter P.F.N., Liu S.Y.C., Chao P.Y., Chen A., Kushida C.A. (2024). Using standardized ultrasound imaging to correlate OSA severity with tongue morphology. Sleep Med..

[7] Liao L.-J., Cho T.-Y., Cheng P.-W., Wang C.-T., Lo W.-C., Huang T.-W. (2016). Submental ultrasonography in diagnosing severe obstructive sleep apnea syndrome. J. Med. Ultrasound.

[8] Page M.J., McKenzie J.E., Bossuyt P.M., Boutron I., Hoffmann T.C., Mulrow C.D., Moher D. (2021). The PRISMA 2020 statement: An updated guideline for reporting systematic reviews. BMJ.

[9] Clark J., Glasziou P., Del Mar C., Bannach-Brown A., Stehlik P., Scott A.M. (2020). A full systematic review was completed in 2 weeks using automation tools: A case study. J. Clin. Epidemiol..

[10] Ouzzani M., Hammady H., Fedorowicz Z., Elmagarmid A. (2016). Rayyan-a web and mobile app for systematic reviews. Syst. Rev..

[11] Kapur V.K., Auckley D.H., Chowdhuri S., Kuhlmann D.C., Mehra R., Ramar K., Harrod C.G. (2017). Clinical practice guideline for diagnostic testing for adult obstructive sleep apnea: An American Academy of Sleep Medicine clinical practice guideline. J. Clin. Sleep Med..

[12] Liu K.-H., Chu W.C., To K.-W., Ko F.W., Tong M.W., Chan J.W., Hui D.S. (2007). Sonographic measurement of lateral parapharyngeal wall thickness in patients with obstructive sleep apnea. Sleep.

[13] Lahav Y., Rosenzweig E., Heyman Z., Doljansky J., Green A., Dagan Y. (2009). Tongue base ultrasound: A diagnostic tool for predicting obstructive sleep apnea. Ann. Otol. Rhinol. Laryngol..

[14] Chen J.W., Chang C.H., Wang S.J., Chang Y.T., Huang C.C. (2014). Submental ultrasound measurement of dynamic tongue base thickness in patients with obstructive sleep apnea. Ultrasound Med. Biol..

[15] Chien C.Y., Chen J.W., Chang C.H., Huang C.C. (2017). Tracking dynamic tongue motion in ultrasound images for obstructive sleep apnea. Ultrasound Med. Biol..

[16] Chen J.W., Huang C.C., Weng C.K., Chang C.H., Wang S.J. (2017). Simultaneous recording of ultrasound and polysomnography during natural sleep in patients with obstructive sleep apnea: A pilot study. J. Sleep Res..

[17] Manlises C.O., Chen J.W., Huang C.C. (2020). Dynamic tongue area measurements in ultrasound images for adults with obstructive sleep apnea. J. Sleep Res..

[18] Hussein S.A., Kamel K.M., Kaddah S.Z., Abd El-Hamid E.E., Shaban M.M. (2020). Role of ultrasonography in assessment of anatomic upper airway changes in patients with obstructive sleep apnea. Adv. Respir. Med..

[19] Chen L., Fang Y., Wang S., Lu Z., Tao J., Lu Y., Nie G. (2021). Ultrasound measurement of upper airway related indicators and patients with obstructive sleep apnea hypopnea correlation study of disease severity. Lin Chuang Er Bi Yan Hou Tou Jing Wai Ke Za Zhi.

[20] Ravindranath S., Ranganath Y.S., Lemke E., Behrens M.B., Marian A.A., Kalagara H., Sadek N., Seering M.S., Wendt L., Eyck P.T. (2025). Correlation of airway POCUS measures with screening and severity evaluation tools in obstructive sleep apnea: An exploratory study. J. Clin. Med..

[21] Weng C.K., Chen J.W., Lee P.Y., Huang C.C. (2017). Implementation of a wearable ultrasound device for the overnight monitoring of tongue base deformation during obstructive sleep apnea events. Ultrasound Med. Biol..

[22] Abuan M.R.A., Lin W.-N., Hsin L.-J., Lee L.-A., Fang T.-J., Chen N.-H., Lo Y.-L., Li H.-Y. (2020). Tongue imaging during drug-induced sleep ultrasound in obstructive sleep apnea patients. Auris Nasus Larynx.

[23] Parekh M.H., Thuler E., Triantafillou V., Seay E., Sehgal C., Schultz S., Keenan B.T., Schwartz A.R., Dedhia R.C. (2023). The application of ultrasound to quantify hyoid motion during drug-induced sleep endoscopy. Laryngoscope.

[24] Parekh M.H., Thuler E., Triantafillou V., Seay E., Sehgal C., Schultz S., Keenan B.T., Schwartz A.R., Dedhia R.C. (2024). Physiologic and anatomic determinants of hyoid motion during drug-induced sleep endoscopy. Sleep Breath..

[25] Chang C.H., Huang C.C., Wang Y.H., Chou F.J., Chen J.W. (2020). Ultrasound shear-wave elastography of the tongue in adults with obstructive sleep apnea. Ultrasound Med. Biol..

[26] Chu C.A., Chen Y.J., Chang K.V., Wu W.T., Özçakar L. (2021). Reliability of sonoelastography measurement of tongue muscles and its application on obstructive sleep apnea. Front. Physiol..

[27] Ontimare Manlises C., Chen J.W., Huang C.C. (2024). A gated recurrent unit model based on ultrasound images of dynamic tongue movement for determining the severity of obstructive sleep apnea. Ultrasonics.

[28] Jones S.E., Aw N., Acord M., Miller S., Sidelnikov D., Haft S.J., Restaino S.M. (2025). Ultrasound predicts drug-induced sleep endoscopy findings using machine learning models. Laryngoscope.

[29] Rodríguez-Alcalá C., Rodríguez-Alcalá L., Ignacio-García J.M., Plaza G., Gozal D., Baptista P., O’Connor-Reina C. (2026). Telemedicine-delivered myofunctional therapy remodels upper airway anatomy in obstructive sleep apnea: A prospective controlled study. J. Clin. Sleep Med..

[30] Saha S., Rattansingh A., Viswanathan K., Saha A., Martino R., Yadollahi A. (2020). Ultrasonographic measurement of pharyngeal-airway dimension and its relationship with obesity and sleep-disordered breathing. Ultrasound Med. Biol..

[31] Terawatpothong A., Sessirisombat C., Banhiran W., Hotokezaka H., Yoshida N., Sirisoontorn I. (2024). Relationship between cephalometric and ultrasonic airway parameters in adults with high risk of obstructive sleep apnea. J. Clin. Med..

[32] Alcala C.R., Alcala L.R., Garcia J.M.I., Plaza G., Baptista P., Lujan G., Mazzei P., Ibañez-Rodriguez J.A., O’connor-Reina C. (2024). Use of ultrasound to verify the impact of telemedicine myofunctional therapy on sleep apnea syndrome: Study protocol proposal. Life.

[33] Pae E.K., Lowe A.A. (1999). Tongue shape in obstructive sleep apnea patients. Angle Orthod..

[34] Fleury B., Rakotonanahary D., Petelle B., Vincent G., Fleury N.P., Meyer B., Lebeau B. (2004). Mandibular Advancement Titration for Obstructive Sleep Apnea: Optimization of the Procedure by Combining Clinical and Oximetric Parameters. Chest.

[35] Hofauer B., Philip P., Wirth M., Knopf A., Heiser C. (2017). Effects of upper-airway stimulation on sleep architecture in patients with obstructive sleep apnea. Sleep Breath..

[36] Huang J., Zhuang J., Zheng H., Yao L., Chen Q., Wang J., Fan C. (2024). A machine learning prediction model of adult obstructive sleep apnea based on systematically evaluated common clinical biochemical indicators. Nat. Sci. Sleep.

[37] Malhotra N., Kedia Y., Goel A.D., Agrawal S., Gupta N. (2025). Ultrasonographic assessment of the airway to evaluate obstructive sleep apnea: A systematic review and meta-analysis. Respir. Med..

[38] Hemrajani P., Dhaka V.S., Rani G., Verma S., Woźniak M., Shafi J., Ijaz M.F. (2025). Integrating physiological signals for enhanced sleep apnea diagnosis with SleepNet. Sci. Rep..

